# Editorial: Rheumatoid arthritis: Pathogenesis and target-treatments

**DOI:** 10.3389/fmed.2023.1145163

**Published:** 2023-02-28

**Authors:** Silvia Piantoni, Sarah Ohrndorf

**Affiliations:** ^1^Rheumatology and Clinical Immunology Unit, Department of Clinical and Experimental Sciences, Azienda Socio Sanitaria Territoriale Spedali Civili and University of Brescia, Brescia, Italy; ^2^Department of Rheumatology and Clinical Immunology, Charité-Universitätsmedizin Berlin, Berlin, Germany

**Keywords:** rheumatoid arthritis, pathogenesis, research, treatments, target

Rheumatoid arthritis (RA) is a chronic autoimmune disease causing progressive articular destructions leading to deformities of the joints. Mainly targeting synovial membranes, it is a systemic inflammatory disease, that affects about 1% of the population (1). The disease strategy of RA is well-established if compared with other rheumatic conditions and has led to improved outcomes (2). Recently, an update of the European recommendations of management has been published (3). Despite the use of many effective immune-targeted drugs, which have dramatically improved the prognosis of the disease during the last years, there are still unmet needs, e.g., accurate biomarkers to stratify patients and to predict therapeutic response (2, 4).

In this Research Topic, we focused on basic research papers presenting novel insights in RA pathogenesis and suggesting potential new molecular targets for therapeutics.

Hernández-Breijo et al. have investigated the role of B cell immunophenotyping to predict remission in RA patients after 6 months of therapy [TNF-inhibitors (TNFi) in combination with methotrexate]. They found out that RA patients who were responsive to TNFi therapy showed a reduction of the relative amount of circulating naïve B cells, that were recently identified as the first B cell subpopulation involved in joint flares (5, 6). As one limitation, it was not possible to show that the reduction of naïve B cells was related to the specific inhibition of the TNF alpha molecule. Therefore, larger studies on RA cohorts treated with different drugs will be needed to confirm the potential utility of the flow cytometric analysis before start of targeted therapies.

Su et al. suggested using the analysis of flow cytometry in the peripheral blood of RA patients. They focused in their analysis on different subtypes of follicular T cells. Compared with healthy controls, RA patients had a significantly higher proportion of follicular helper-like T cells, whilst the amount of regulatory T cells was reduced. The authors concluded that T cells might represent potential circulating biomarkers in RA, especially since they play a central role in the pathogenesis of the disease in the early phase. Furthermore, they are responsible for maintenance of the autoimmune process as well as their activation requires co-stimulatory signals provided by surface molecules on the membrane (7).

Another paper of our Research Topic has dealt with the T cell related gene polymorphisms in RA. Liu et al. combined an original case-control study on a Chinese population with a meta-analysis focusing on genes. The genes of interest were CTLA-4, CD80/86, and CD28, as being related to co-stimulatory mechanisms. Interestingly, they found out that some CTLA-4 polymorphisms decreased the risk of RA development, furthermore, the CTLA-4 molecule is central in the co-inhibition toward early T cell activation, restraining immune response (7).

Studying synovial tissues with advanced and combined laboratory techniques, El Shikh et al. demonstrated new insights into the regulation of follicular dendritic cells. Specific molecules regulate the differentiation of these cells influencing fibroid or a lymphoid synovitis, which are related to prognosis in RA. This study revealed the interactions among new molecules that could potentially be involved in the therapy for difficult-to-treat RA patients.

Finally, Shi et al. suggested a potential drug target, which has already been investigated in the context of cancer research. The authors used an arthritis model to demonstrate the role of METTL3 (methyltransferase-like 3), a component of the N^6^-methyladenosine methyltransferase complex and a regulator of posttranscriptional processes, in the development of the human disease. They presented an upregulation of METTL3 in RA fibroblast-like synoviocytes both in human and murine synovial tissues, enhancing their capability to proliferate, invade, migrate and, also, to promote the production of pro-inflammatory cytokines.

Taken together, the results of these studies open new research areas in the field of RA pathogenesis.

Basic science studies exist, that exploit the current advanced technologies for the better understanding of the functional mechanisms of synovitis. They usually make use of invasive mechanisms such as synovial biopsies, as recently demonstrated by a precision-medicine, randomized clinical trial (8). In fact, the integration of molecular pathology signatures into clinical algorithm is a convincing future perspective. We also want to encourage basic researchers to further develop translational studies in RA.

In future, the combination of an integrated approach including laboratory-based studies, clinical scores and patient reported outcomes as well as personalized digital medicine tools has the potential to overcome the current unmet needs in RA.

## Author contributions

SP wrote the first draft. SO edited and reviewed the draft. All authors approved the final version of the manuscript.
